# An evaluation of quality of sleep and prevalence of sleep disorders among adult population of Urban slum of Bangalore - a cross sectional study

**DOI:** 10.12688/f1000research.140184.1

**Published:** 2023-10-31

**Authors:** Mithun Rao, Shobha Bhushan, Ranganath TS

**Affiliations:** 1 Kasturba Medical College, Mangalore, Manipal Academy of Higher Education, Manipal, India; 2 Bangalore Medical College and Research Institute, Bangalore, India

**Keywords:** Sleep, Adult, Quality of sleep, Sleep Hygiene, Sleep disturbance.

## Abstract

**Background:** Sleep can be defined as a state of reduced attention from where the person can be woken up by any kind of stimuli. Sleep difficulties are a major group of disorders affecting one third of the adult population. The present study was taken up to assess the sleep quality and prevalence of sleep disorders among the adult population in the urban slum area of H Siddaiah Road Urban Primary Health Center (UPHC), which is in the Urban Field Practice Area, BMCRI.

**Methods:** Stratified random sampling was used to select 821 adults in the population of 18-60 years of age. Ethical clearance was obtained from the Institutional Ethical Committee. A pretested semi-structured questionnaire was used to interview the adults after obtaining their consent. The data was entered in Microsoft Office Excel and analysed using SPSS ver20.0.

**Results:** The study population was 52.81% females and 77.5% in the age group of 18-30 years. Most of the study population were Hindus (78.90%), and only 3.8% of the study population were illiterate. Most of them were employed (86.12%).

Substance use was present in 82.9% of the study participants and overcrowding was present in 51.3% of the subjects. Female gender, being unemployed, living with relatives, overcrowding, and substance use such as alcohol and smokeless tobacco were the factors associated with poor sleep quality as measured using the Pittsburgh Sleep Quality Index. Among the study participants having poor sleep quality, most of participants needed further clinical assessment for insomnia (86%) followed by assessment for sleep apnoea (50.5%).

**Conclusions:** 200 (24.36%) study participants were determined to have poor sleep quality. Gender, marital status and overcrowding were the factors associated with poor sleep quality. A significant number of study participants need further assessment on insomnia, sleep apnoea and psychiatric disorders.

## Introduction

Sleep is defined as unconsciousness from which the person can be aroused by sensory or other stimuli. Sleep is the vital component of health and is essential for mental and physical wellbeing and crucial for rejuvenation of body. ^1^ Sleep is a recurrent state of reduced attention to the surrounding environment, which is essential to maintain and restore body functions, memory and cognitive performance. Sleep plays a crucial role in maintenance of normal functioning of many systems including endocrine and immune systems. ^2^ The average adult needs 7 to 9 hours of excellent sleep per day, ^3^ which translates to sleeping for nearly one-third of their life. Poor sleep can have detrimental impacts on one’s mental and physical health. ^3^ Theoretically sleep is composed of two physiological phases, namely non-rapid eye movement (NREM) sleep and rapid eye movement (REM) sleep. ^1^

Adults frequently experience problems with their ability to sleep. According to previous studies, 10 to 40 percent of adults experience different types of sleep disturbances. ^4^^,^^5^ Sleep issues can significantly lower people’s quality of life. Sleep hygiene and sleep problems are not well understood by the general public. One of the most prevalent sleep problems is insomnia, which is also a sign of poor mental health in the elderly population. Subjective complaints of trouble falling asleep, staying asleep, and early morning awakenings are its hallmarks. This has serious daytime effects such weariness, low energy, difficulties with cognition (attention and memory), and mood disorders, which can cause serious distress and functional deficits, especially as people age. ^6^

A wide range of detrimental health effects, such as an increased risk of hypertension, diabetes mellitus, obesity, immunological deficiency, coronary heart disease, and stroke, are linked to poor sleep quality. ^6^ Conversely, people who have any of these illnesses are more likely to experience sleep-related issues. People who struggle with sleep disturbances have much greater rates of using drugs, low quality of life, depressed mood, anxiety, and panic attacks. These comorbidities will affect an older population more frequently. ^7^

Good sleep quality is associated with wide range of positive outcomes such as better health, less daytime sleepiness and better psychological functioning. Many factors contribute to sleep patterns, including sleep hygiene practices. ^8^ Sleep hygiene is the variety of different practices and habits that are necessary to have good nighttime sleep quality and full daytime alertness. ^9^ Regular exercise, regular bedtimes and wake-up times, and no midday naps are all behaviors that promote sleep. The use of stimulants like caffeine or cigarettes, participating in stimulating or unpleasant activities right before bed, and using alcohol are all actions that prevent people from falling asleep.

In the history of humanity, sleep issues are a relatively new development. Chronic sleep deprivation was a very uncommon issue for the majority of humanity before the discovery and widespread use of electricity and artificial light. ^10^

Though there have been studies conducted in the aged population and students in other countries about the quality of sleep and its associated factors, there has been lack of literature on quality of sleep among the adult population and its associated risk factors in India, especially in urban slums of India. Hence the present study has been taken up to assess the quality of sleep and prevalence of sleep disorders among the adult population in the urban slum which is the Urban Field Practice Area of the Department of Community Medicine, Bangalore Medical College and Research Institute (BMCRI), Bengaluru.

### Objectives

1. To assess the quality of sleep and the factors associated with it among the adult population of the Urban Field Practice Area of BMCRI.
2. To estimate the prevalence of sleep disorders among them.

## Methods

### Study design

This study had a cross-sectional design. The study took place between November 2018 and May 2020.

### Study location

The study was conducted at the urban slum area of H Siddaiah Road Urban Primary Health Center (UPHC), which is in the Urban Field Practice Area of BMCRI. Being one of the largest slums in Bangalore, it represents the urban slums of Southern India.

### Study population

The study population was adults belonging to the age group between 18 and 60 years and residing in the Urban slum of Bangalore.

### Inclusion criteria

1. Age 18 to 60 years and is a permanent resident (residing for more than six months in the area) of the area.
2. The individuals who are present in the area at the time of interview.
3. People who are willing to give consent for the study.

### Exclusion criteria

1. People who are not willing to give consent for the study.
2. Those who are severely ill and/or diagnosed with a mental health condition.
3. Lactating women.

### Ethics and consent

The IEC (Institutional Ethics Committee) of Bangalore Medical College and Research Institute, Bangalore has reviewed the study and has granted approval prior to the onset of the study (BMC/PG/124/2018-19, on 3 ^rd^ January 2018). Study subjects had the purpose of the study explained to them and written informed consent was obtained before proceeding. Confidentiality of the present study data was maintained in accordance with the Declaration of Helsinki.

### Sample size and participant selection

There are 3 sectors in the urban field practice area; the total population is around 30,500 and the total number of households is 6045. The study size calculation was conducted by probability proportionate to size sampling (PPS). The sample size for each sector will be derived from formula:

$$n1=\frac{\text{Household of each sector × sample size}}{\text{Total number of households}}$$

Example 1st sector has a household of 1712, so,

$$n1=\frac{1712\times 820}{6045}=232.23=233$$

The same formula will be applied for other sectors, thus making a total sample size of 821.

Systematic random sampling is used for the selection of the study population. After calculating the required number of participants from each sector, a random number is generated and the corresponding household is visited for the interview. The next household is visited by adding the sample interval to the randomly generated number and the interview is continued till we reach the required target. Only one adult member from each household was selected for the interview.

Taking the example of the 1st sector,

Sample interval is calculated by as using the formula.

$$\text{Sample interval}=\frac{1712}{233}=7.34$$

Let the random number generated be ‘3’. The third household of the sector is visited and interviewed. The next household number to be visited will be 3 + 7 = 10th house (random number + sample interval). If there is a locked house, the adjacent house to the locked house is interviewed. Like this the interview is continued till the adequate sample size is reached.

### Data collection

After obtaining the written informed consent and assuring confidentiality to the study participants, information regarding the sociodemographic factors and the details regarding the quality of sleep were collected, using pre-tested, semi-structured questionnaires (Pittsburgh Sleep Quality Index and Sleep Disorders Questionnaire). ^11^^,^^12^ The data collection was done by the researcher by visiting the houses in the community for a duration of 6 months. The study participants were interviewed using the questionnaires described above as well as a sociodemographic questionnaire.

### Overview of questionnaires

*Pittsburgh Sleep Quality Index (PSQI)*

The PSQI is an instrument for the assessment of outcome of sleep quality over the preceding one month which helps in categorizing the subjects based on the quality of sleep. This tool is designed to evaluate self-rated sleep quality.

The questionnaire evaluates sleep quality over the past one month with the help of 19 items, which generate seven component scores namely subjective sleep quality, sleep latency, sleep duration, habitual sleep efficiency, sleep duration, use of sleeping medication and daytime dysfunction. The sum of the component scores yields a global score. It first contains 4 open questions, following which there are questions with 4-point scale. Each component score ranges from 0 to 3 (‘no difficulty’ to ‘severe difficulty’). The global score can range from ‘0’ to ‘21’ and a score >5 suggested poor sleep quality. This questionnaire requires around 10 minutes for completion. The PSQI has adequate psychometric properties. A PSQI tool had sensitivity of 89.6% and 86.5% specificity. The test-retest reliability with scores can be correlated to the polysomnographic results.

*Sleep Disorders Questionnaire*

This is a questionnaire developed by Toward Optimized Practice Group to assist physicians in the evaluation of sleep disorders. It helps clinicians to know to screen for sleep related disorders; thus, helping in treating much earlier in primary care settings and early referral where it is needed. The target population of this questionnaire is adults, hence children were excluded from the study.

### Analysis

Data collected was then analyzed with SPSS version 20. Results have been expressed in means, proportions and standard deviations. Appropriate parametric and non-parametric tests are applied wherever necessary. Chi square test was used to measure the association between quality of sleep and the socio demographic variables. The student’s t test was used to measure the statistically significant difference among the gender with respect to quality of sleep.

## Results

### Demographic profile

Among 821 study subjects, 432 were females and 389 were males. 636 (77.5%) study participants belonged to the age group of 18-30 years ( Table 1). ^17^ The mean age of the participants was 30.86 ± 9.25 years among males and 28.92 ± 7.37 years among females. The majority of the population (648; 78.9%) were following Hindu religion, and 102 (12.4%) followed Christian religion. Among the study subjects, only 31 (3.8%) were illiterate, the rest all were literate.

**Table 1.** Distribution of study subjects according to age and sex (n = 821).

Age group (years)	Female (n = 432) N (%)	Male (n = 389) N (%)
**18-30**	355 (43.24%)	281 (34.23%)
**31-40**	38 (4.63%)	48 (5.85%)
**41-50**	29 (3.53%)	37 (4.51%)
**51-60**	10 (1.22%)	23 (2.79%)
**Total**	**432 (52.62%)**	**389 (47.38%)**

707 (86.12%) study participants were employed. Among the total participants surveyed, 482 (58.7%) were unmarried. 329 (40.1%) study participants were living with their parents. 411 (50.1%) study participants were living in a nuclear family, whereas 383 (46.4%) study participants were living in a joint family. Overcrowding was present in 421 (51.3%) houses ( Table 2).

**Table 2.** Baseline characteristics of study population (n=821).

Variable	Category	N (%)
**Religion**	**Hindu**	648 (78.90%)
	**Christian**	102 (12.50%)
	**Muslim**	71 (8.6%)
**Literacy status**	**Illiterate**	31 (3.80%)
	**Primary school**	4 (0.50%)
	**Middle school**	98 (11.90%)
	**High school**	291 (35.40%)
	**PUC/intermediate/PUC**	80 (9.70%)
	**Graduate**	261 (31.80%)
	**Professional**	56 (6.80%)
**Employment status**	**Unemployed**	114 (13.88%)
	**Employed**	707 (86.12%)
**Marital status**	**Married**	327 (39.8%)
	**Separated/widow/widower**	12 (1.4%)
	**Unmarried**	482 (58.7%)
**Living arrangement**	**Living Alone**	94 (11.4%)
	**Living with children**	20 (2.4%)
	**Living with parents**	329 (40.1%)
	**Living with relatives**	118 (14.4%)
	**Living with spouse**	60 (7.3%)
	**Living with spouse and children**	200 (24.4%)
**Type of family**	**Joint family**	381 (46.40%)
	**Nuclear family**	411 (50.10%)
	**Three generation family**	29 (3.50%)
**Overcrowding**	**Present**	421 (51.3%)
	**Absent**	400 (48.7%)
**Substance Use**	**Present**	681 (82.90%)
	**Absent**	140 (17.10%)

681 (82.9%) participants reported substance use of any form. Of 681 participants, 520 (63.3%) of them were consuming coffee/tea, whereas 224 (27.3%) were using smokeless tobacco. 199 (24.2%) of the study population were smokers and 148 (18.0%) were consuming.

Based on the global PSQI scores, study participants were divided into two groups. Those who had a global PSQI score of less than or equal to 5 were categorized as having good sleep quality (621, 75.63%). Participants who had a global PSQI score of more than 5 were categorized as having poor sleep quality (200, 24.37%) ( Table 3).

**Table 3.** Distribution of study subjects based on quality of sleep (n = 821).

Quality of sleep	N (%)
**Good sleep (PSQI ≤ 5)**	621 (75.63%)
**Poor sleep (PSQI > 5)**	200 (24.37%)

The association was measured using the Chi square test between the quality of sleep and various socio-demographic factors, which have been mentioned above. A statistically significant association (p value < 0.05) was found between quality of sleep and gender (p value = 0.03), marital status (p value = 0.005), living arrangements (p value = 0.000), type of family (p value < 0.0001) and presence of overcrowding (p value < 0.0001) ( Table 4).

**Table 4.** Association between quality of sleep and socio-demographic factors.

		Good sleep (n=621)	Poor sleep (n=200)	χ ^2^ Value	p value
**Gender**	**Female**	309 (71.53%)	123 (28.47%)	**8.365**	**0.003** ^*^
	**Male**	312 (80.21%)	77 (19.79%)		
**Employment status**	**Unemployed**	87 (76.32%)	27 (23.68%)	**0.033**	**0.907**
	**Employed**	534 (75.53%)	173 (24.47%)		
**Marital status**	**Married**	264 (80.73%)	63 (19.27%)	**12.788**	**0.005** ^*^
	**Separated/widow/widower**	12 (100.00%)	0 (0.00%)		
	**Unmarried**	345 (71.58%)	137 (28.42%)		
**Living arrangement**	**Living Alone**	75 (79.79%)	19 (20.21%)	**29.455**	**<0.001** ^*^
	**Living with children**	20 (100.00%)	0 (0.00%)		
	**Living with parents**	233 (70.82%)	96 (29.18%)		
	**Living with relatives**	78 (66.10%)	40 (33.90%)		
	**Living with spouse**	43 (71.67%)	17 (28.33%)		
	**Living with spouse and children**	172 (86.00%)	28 (14.00%)		
**Type of family**	**Joint family**	286 (75.07%)	95 (24.93%)	**29.455**	**<0.001** ^*^
	**Nuclear family**	320 (77.86%)	91 (22.14%)		
	**Three generation family**	15 (51.72%)	14 (48.28%)		
**Substance use**	**Present**	507 (74.45%)	174 (25.5%)	**3.070**	**0.084**
	**Absent**	114 (81.43%)	26 (18.57%)		
**Overcrowding**	**Present**	247 (58.67%)	174 (41.33%)	**135.044**	**<0.001** ^*^
	**Absent**	374 (93.50%)	26 (6.50%)		

Study participants who had poor sleep quality (200) were further subjected to the sleep disorders questionnaire. Based on their response to the sleep disorders questionnaire, study participants were further categorized into various sleep related disorders. Among those (200), insomnia was observed in 82.5%, sleep apnoea in 50.5%, psychiatric disorders in 39.5%, parasomnias in 36.5%, circadian rhythm disorders in 35.5%, and movement disorders in 8%, which may need further clinical evaluation ( Table 5).

**Table 5.** Distribution of study subjects based on the response to the sleep disorders questionnaire.

Diagnostic Domain	Presence of sleep disorders N (%)
**Insomnia**	165 (82.5%)
**Psychiatric disorders**	79 (39.5%)
**Circadian rhythm disorder**	71 (35.5%)
**Movement disorders**	16 (8%)
**Parasomnias**	73 (36.5%)
**Sleep apnoea**	101 (50.5%)

## Discussion

The present study evaluates the quality of sleep and its related factors along with assessing the prevalence of sleep disorders in the urban slum area of H Siddaiah Road Urban Primary Health center (UPHC), which is the Urban Field Practice Area of BMCRI. The present study explores the importance of various factors affecting the sleep quality and the prevalence of sleep disorders among the adult population of the urban slums. The findings are based on interviews of the 821 participants at the field level.

### Sociodemographic characteristics of the study subjects

Among the 821 subjects, more than half the population is female, 432 (52.6%) and the majority belonged to the age group of 18-30 years, (636; 77.5%) with a mean age of 30.86 ± 9.258 years among males and 28.92 ± 7.373 years among females. A community based cross-sectional study conducted by Berhanu H *et al.* in Ethiopia had a preponderance of males (60.9%), with mean age of the total population of 38.7 + 12.5 years. ^13^ A similar study conducted by Madrid-Valero JJ *et al.* in Spain had 54.7% females with a mean age of the study participants being 53.7 years. ^14^ Yet another study conducted by Panda S *et al.* in South India had an even gender distribution (M:F 29:21), with the mean age of the respondents being 35.1±8.7 years having mostly southern India representation. ^15^

As the study by Berhanu H *et al.* was in the similar set up as the present study, the socio demographic characteristics were quite similar, whereas the study by Madrid-Valero JJ *et al.* had a higher mean age, which may be due to the different methodology which was followed. ^13^^–^^14^

### Factors affecting quality of sleep

In the present study, the participants went to bed on average at 10.20 PM with a sleep latency of 15.16, minutes and the total duration of sleep experienced by them was 7.8 hours. While in the study conducted by Berhanu H *et al.*, participants reported an average night’s sleep duration of 6.8 ± 2.1 hours, with the study participants having gone to bed at 10.20 PM. ^13^ One more study conducted by Madrid-Valero JJ *et al.* had assessed the mean sleep duration during night time which was found to be 6.43 + 1.3 hours, and sleep latency was not assessed in the study. ^14^ In the study conducted by Panda S *et al.*, average time-to-fall-asleep was 22 minutes (range: 5-90 min), and mean duration of actual sleep was 7 hours (range: 3.5-9.1 hours). ^15^

All the studies had similar results with respect to total duration of night sleep experienced.

In the present study, about one fourth of the study participants (24.36%) reported having poor sleep quality (global PSQI > 5), whereas the study conducted by Panda S *et al.* had a majority (93.8%) of the population reporting good-quality sleep (global PSQI ≤ 5). In the study conducted by Berhanu H *et al.*, 4% participants were assessed as poor sleepers by a global PSQI score greater than 5. A similar study done by Madrid-Valero JJ *et al.* showed 38.2% of the subjects having poor sleep quality (global PSQI > 5). ^13^^–^^15^

In the present study, it was seen that female gender, being unmarried, overcrowding and use of substance such as alcohol and tobacco were having a significant impact on the quality of sleep. The study conducted by Berhanu H *et al.* also reported similar results with female gender, having less monthly income, Khat chewing practices and consumption of alcohol being correlated factors for poor quality of sleep. ^13^

The present study showed that further evaluation is needed, as insomnia was reported in 82.5% of the 20 participants with poor sleep quality, sleep apnoea in 50.5%, psychiatric disorders in 39.5%, parasomnias in 36.5%, circadian rhythm disorders in 35.5% and movement disorders in 8%. Another study conducted by Ram S *et al.* ^16^ in USA, prevalence of sleep disorders was highest for sleep apnoea (4.2%), followed by insomnia (1.2%) and RLS (0.4%). The reported rates of sleep related disorders varied between 20.0% and 34.2% depending on the instrument used in the questionnaire. In the study conducted by Panda S *et al.*, insomnia, sleep-related breathing disorders, narcolepsy, and restless legs syndrome (RLS) were reported to be 18.6%, 18.4%, 1.04% and 2.9% respectively. ^14^^–^^16^

## Conclusion

The present study indicated that one fourth of the adult population residing in the Urban Field Practice Area of Community Medicine Department, BMCRI reported poor sleep quality. It was observed that the poor quality of sleep is associated with various factors such as gender, marital status, living arrangement, type of family and overcrowding. The present study also observed that among the people having poor sleep quality, insomnia was seen in four fifths, sleep apnoea in half, and psychiatric disorders in two fifths of the study population. They need further evaluation to confirm the sleep disorders.

## Data Availability

figshare: Masterchart.xlsx. https://doi.org/10.6084/m9.figshare.23997591.v1 ^17^ figshare: Coding key.xlsx. https://doi.org/10.6084/m9.figshare.23997585.v1 ^18^ figshare: Questionnaire.docx. https://doi.org/10.6084/m9.figshare.23997693.v1 ^19^ Data are available under the terms of the Creative Commons Attribution 4.0 International license (CC-BY 4.0).
