# Supplementary material for: Poor quality of sleep and it's associated factors among urban slum-dwellers of Bengaluru- a cross-sectional study
Source: F1000Res. 2024 May 17;12:1426. Originally published 2023 Oct 31. [Version 3] doi: 10.12688/f1000research.140184.3 (PMC11053344; doi:10.12688/f1000research.140184.3)
Supplement: Peer review history

# Reviewer report: Reviewer response for version 2

**DOI:** 10.5256/f1000research.163547.r260733

**Published:** 2024-04-26

**Authors:** Sudhir Prabhu

**Affiliations:** 1 Department of Community Medicine, Father Muller Medical College, Mangalore, India

"minor comments"

 1. "Was this a facility based or community based study? to understand who constituted the study universe and was there a fair representation of the urban slum dwellers as possible research study subjects. This might give better clarity the the readers and scientific community when this paper is being read or used for evidence.

 2. Was the study tool standardized for local context and administered after pre testing in a pilot population for ensuring internal validity ?

  3. The study mentions 24% of the study population had poor sleep quality, being a cross sectional study this could be one of the limitations of the research work (ongoing social and medical issues could influence the sleep quality)

 4. was the influence of these clinico social factors considered for multivariate analysis?" use of summary statistics like Odds ratio or adjusted OR with 95% CI may be useful in understanding the potential role  of risk factors under evaluation

 Summary :

 overall its a good and novel study as poor quality of sleep has been linked to various social and economical consequences apart from health related adverse events. Studying it on a selected set of community which are vulnerable (slum dwellers) makes it even more important for health care professionals and public health researchers.

Is the work clearly and accurately presented and does it cite the current literature?

Yes

If applicable, is the statistical analysis and its interpretation appropriate?

Yes

Are all the source data underlying the results available to ensure full reproducibility?

Yes

Is the study design appropriate and is the work technically sound?

Yes

Are the conclusions drawn adequately supported by the results?

Yes

Are sufficient details of methods and analysis provided to allow replication by others?

Yes

Reviewer Expertise:

Public health

I confirm that I have read this submission and believe that I have an appropriate level of expertise to confirm that it is of an acceptable scientific standard.

# Reviewer report: Reviewer response for version 2

**DOI:** 10.5256/f1000research.163547.r254562

**Published:** 2024-03-18

**Authors:** Pracheth Raghuveer

**Affiliations:** 1 Department of Epidemiology, National Institute of Mental Health and Neurosciences, India, Bengaluru, Karnataka, India

The authors have addressed all the queries and comments. I hereby approve the manuscript without any reservations.

Is the work clearly and accurately presented and does it cite the current literature?

Partly

If applicable, is the statistical analysis and its interpretation appropriate?

Yes

Are all the source data underlying the results available to ensure full reproducibility?

Yes

Is the study design appropriate and is the work technically sound?

Yes

Are the conclusions drawn adequately supported by the results?

Yes

Are sufficient details of methods and analysis provided to allow replication by others?

Yes

Reviewer Expertise:

Mental Health, Geriatrics, Non-Communicable Diseases, Maternal and Child Health, Neuro Sciences, Medical Education

I confirm that I have read this submission and believe that I have an appropriate level of expertise to confirm that it is of an acceptable scientific standard.

# Reviewer report: Reviewer response for version 1

**DOI:** 10.5256/f1000research.153515.r219855

**Published:** 2024-01-24

**Authors:** Pracheth Raghuveer

**Affiliations:** 1 Department of Epidemiology, National Institute of Mental Health and Neurosciences, India, Bengaluru, Karnataka, India

Overall, it is an important paper that  highlights an under-diagnosed issue of sleep. The sample size  covered is 821 which is remarkable.  It is a community-based study that may be generalizable to the urban slum dwelllers of Bengaluru.  I congratulate the team of authors for a commendable job. However, there is scope for improvement to make it an even better draft. There are a few gaps that may have to be plugged before it can be  accepted.

 1.  The title may be modified to" Poor quality of sleep and it's associated factors among urban slum-dwellers of Bengaluru- a cross-sectional study" as this was the primary objective/ aim. 

 2. In the methods, please mention the number of items, cut-offs and information about validity of the Sleep Disorders Questionnaire. 

 3. In the results, "Factors affecting quality of sleep" may be modified to "factors associated" as the analysis looks at associations.

 4. In the results, it has been mentioned that 39.5% had psychiatric disorders. How was this assessment carried out? Did the Sleep  Disorders Questionnaire also assess mental health problems.  Please justify. 

 4.  Discussion needs strengthening. Kindly mention few  strengths and limitations of the study in the discussion.

Is the work clearly and accurately presented and does it cite the current literature?

Partly

If applicable, is the statistical analysis and its interpretation appropriate?

Yes

Are all the source data underlying the results available to ensure full reproducibility?

Yes

Is the study design appropriate and is the work technically sound?

Yes

Are the conclusions drawn adequately supported by the results?

Yes

Are sufficient details of methods and analysis provided to allow replication by others?

Yes

Reviewer Expertise:

Mental Health, Geriatrics, Non-Communicable Diseases, Maternal and Child Health, Neuro Sciences, Medical Education

I confirm that I have read this submission and believe that I have an appropriate level of expertise to confirm that it is of an acceptable scientific standard, however I have significant reservations, as outlined above.

# Response

**Published:** 2024-03-08

**Authors:** Mithun Rao

**Affiliations:** Community Medicine, Kasturba Medical College Mangalore, Mangalore, Karnataka, India

Point to point response to Reviewer's comments:  

**Dr. Pracheth Raghuveer**

 1. **Comment: **The title may be modified to" Poor quality of sleep and it's associated factors among urban slum-dwellers of Bengaluru- a cross-sectional study" as this was the primary objective/ aim. **Author’s Response: **We agree with your comment. As per the suggestion title has been modified.

**Action Taken in the manuscript: **As per the suggestion title has been modified in the manuscript.

 2. **Comment: **In the methods, please mention the number of items, cut-offs and information about validity of the Sleep Disorders Questionnaire.

**Author’s Response: **We respect the comments. As per the suggestion following details have been added.

**Action Taken in the manuscript: **The details added are mentioned below.

 Sleep Disorders Questionnaire has 16 items in total. Each item has a grading scale from 1-5 with 1 being never, 2-rarely, 3- occasionally, 4-most nights/days and 5 – always.

 Diagnostic Domains of Sleep Disorders Questionnaire is as follows:

 1) Insomnia: Q1-5

 2) Psychiatric Disorders: Q6-9

 3) Circadian Rhythm Disorder: Q10

 4) Movement disorders: Q11-12

 5) Parasomnias Q13

 3. **Comment: **In the results, "Factors affecting quality of sleep" may be modified to "factors associated" as the analysis looks at associations.

**Author’s Response: **Thank you for the comment Sir. As per the suggestion, we shall modify the wordings.

**Action Taken in the manuscript: **"Factors affecting quality of sleep" phrase mentioned in the results part of the manuscript has been modified to "factors associated" as the details deals with association.

 4. **Comment: **In the results, it has been mentioned that 39.5% had psychiatric disorders. How was this assessment carried out? Did the Sleep  Disorders Questionnaire also assess mental health problems.  Please justify.

**Author’s Response: **Thank you for the query Sir. The Sleep  Disorders Questionnaire screens for the mental health problem.

**Action Taken in the manuscript: **The following detail has been added under methodology which helps in better understanding.

 Diagnostic Domains of Sleep Disorders Questionnaire:

 1) Insomnia: Q1-5

 2) Psychiatric Disorders: Q6-9

 3) Circadian Rhythm Disorder: Q10

 4) Movement disorders: Q11-12

 5) Parasomnias Q13

 5. **Comment: **Discussion needs strengthening. Kindly mention few strengths and limitations of the study in the discussion.

**Author’s Response: **Thank you for the suggestions Sir. We shall add the strengths and limitations as per the suggestion given Sir.  

**Action Taken in the manuscript: **The following are the strengths and limitations.

 Strength:

1. This study is one of the kind among Urban slum dwellers of a metropolitan city.
2. Study participants belong to a category of socially vulnerable population.
3. The sample size of the study is more than 800 making it a large sample size.

Limitations:

1. Since Bangalore Urban slum dwellers have unique characteristics in comparison to slum dwellers of metropolitan city, the results may not be uniform across the cities.
2. As the data collection used to take place during duty hours, working population would have been missed.
